# A Prospective Observational Study to Assess the Diagnostic Accuracy of Suprasternal Versus Subxiphoid Ultrasonography for Endotracheal Intubation in Patients Undergoing Elective Surgery

**DOI:** 10.7759/cureus.87594

**Published:** 2025-07-09

**Authors:** Deepak Vijaykumar Kadlimatti, Lekhashree BK, Amithkumar SK, Salim M Iqbal, Kesa Sahithi Balamreddy

**Affiliations:** 1 Department of Anaesthesia, Dr. B.R. Ambedkar Medical College and Hospital, Bangalore, IND

**Keywords:** etco2, et tube position, prospective study, subxiphoid ultrasonography, suprasternal ultrasonography

## Abstract

Objective: This study compared the diagnostic accuracy and time efficiency of suprasternal versus subxiphoid ultrasonography for endotracheal tube (ETT) confirmation.

Methodology: A prospective observational study was conducted on 50 patients classified as American Society of Anesthesiologists Physical Status (ASA-PS) 1 or 2 who were scheduled for elective surgical procedures under general anesthesia. After endotracheal intubation (ETI), tube placement was verified using three methods: suprasternal ultrasonography, visualizing the characteristic “bullet sign”; subxiphoid ultrasonography, assessing diaphragmatic motion; and continuous waveform capnography. The time required for confirmation and the diagnostic accuracy of each method were systematically recorded.

Results: Suprasternal ultrasound was significantly faster (5.58 ± 1.14 seconds) than capnography (31.50 ± 4.84 seconds) (p < 0.001) and auscultation (33.38 ± 4.58 seconds) (p < 0.001). Subxiphoid ultrasound took 20.32 ± 4.60 seconds (p < 0.001). No false positives or false negatives were observed. Both ultrasound methods showed 100% agreement with capnography.

Conclusion: Suprasternal and subxiphoid ultrasonography are equally accurate but faster than capnography for ETT confirmation in low-risk surgical patients. Due to its speed, suprasternal ultrasound may be preferable in time-critical scenarios.

## Introduction

Endotracheal intubation (ETI) is a critical procedure performed in operating rooms and emergency settings to secure the airway and ensure adequate ventilation [1]. Confirming the correct placement of the endotracheal tube (ETT) is essential, as inadvertent esophageal intubation can lead to hypoxia, brain injury, or even death [2]. Traditional methods for verifying ETT placement include auscultation, capnography, and chest radiography. However, these techniques have limitations, including subjectivity, delays in confirmation, and occasional false results [3].

Point-of-care ultrasonography (POCUS) has recently emerged as a rapid, non-invasive, and reliable alternative for confirming ETT placement [4]. Ultrasonography offers real-time visualization of the ETT, reducing the time to confirmation and improving patient safety. The suprasternal and subxiphoid views are two primary ultrasound approaches for assessing ETT placement. The suprasternal approach visualizes the trachea and adjacent structures in the neck, while the subxiphoid approach assesses diaphragmatic and lung movement [5]. Both methods have shown promise, but comparative data on their diagnostic accuracy and time efficiency remain limited.

The suprasternal view allows direct visualization of the ETT within the trachea, with studies reporting high sensitivity and specificity for detecting tracheal intubation [6]. However, this approach may be challenging in patients with a short neck, obesity, or cervical spine injuries. In contrast, the subxiphoid approach evaluates bilateral lung sliding and diaphragmatic movement, indirectly confirming tracheal intubation [7]. While this method is less operator-dependent for lung sliding assessment, it may be less specific in cases of unilateral lung pathology or poor sonographic windows.

Despite the growing adoption of ultrasound for ETT confirmation, there is no consensus on the optimal approach. Previous studies have evaluated these methods independently, but a direct comparison of their diagnostic accuracy and time efficiency is lacking [8]. Identifying which method provides faster and more reliable confirmation can improve clinical decision-making, particularly in time-sensitive scenarios such as emergency intubation or critical care settings.

This prospective observational study aimed to compare the diagnostic accuracy of suprasternal versus subxiphoid ultrasonography for confirming endotracheal intubation in patients undergoing elective surgery. Additionally, the study assessed the time required for ETT confirmation using both approaches. We evaluate these parameters to determine which ultrasound method is more efficient and reliable for routine clinical use. The findings may contribute to standardized guidelines for ultrasound-assisted ETT confirmation, improving patient outcomes and reducing complications associated with misplaced intubation. Given the clinical urgency of promptly verifying correct endotracheal tube placement, particularly in high-risk or time-sensitive situations, this study sought to address effectiveness and efficiency. The primary objective was to assess diagnostic accuracy, while the secondary objective focused on comparing the time efficiency of suprasternal versus subxiphoid ultrasonography. We hypothesized that both approaches would demonstrate high accuracy, but the suprasternal method would enable faster confirmation due to its direct visualization of tracheal structures.

## Materials and methods

Study design

This prospective observational study was conducted following approval from the Institutional Ethics Committee of Dr. B.R. Ambedkar Medical College and Hospital (approval number: EC 449) and registration with the Clinical Trial Registry of India (CTRI/2024/03/064852).

Eligibility criteria

The study initially enrolled 56 patients aged 18-80 years, classified as American Society of Anesthesiologists Physical Status (ASA-PS) 1 or 2, who were scheduled for elective surgery under general anesthesia requiring endotracheal intubation. Six patients were excluded due to anticipated difficult intubation by the predefined exclusion criteria. The remaining 50 patients met the inclusion criteria and were included in the final analysis. Exclusion criteria comprised patients with abnormal airway anatomy, anticipated difficult intubation, or a body mass index (BMI) greater than 30 kg/m².

Airway assessment criteria

Airway assessment was conducted during the pre-anesthetic evaluation performed 24 hours before surgery. Mouth opening was measured in centimeters, and patients with an inter-incisor distance of less than 3 cm were excluded. The modified Mallampati classification was assessed with the patient seated, mouth fully open, and tongue maximally protruding; only patients classified as Mallampati grade 1 or 2 were included. The upper lip bite test (ULBT) assessed mandibular mobility and dental occlusion. Patients with grade 3 on the upper lip bite test were excluded from the study.

Sample size calculation

The sample size was determined based on a 2022 study by Mousavi et al. [9] using the following formula:

\[ n = \frac{Z_{\alpha}^2 \cdot \text{Sn} \cdot (100 - \text{Sn})}{d^2} \]

where $Z_{\alpha} = 1.96$ (95% confidence interval (CI)), $\text{Sn} = 80$ (sensitivity), and $d = 12$ (precision, which is 15% of sensitivity). Substituting the values:

\[ n = \frac{2 \cdot (1.96)^2 \cdot 80 \cdot (100 - 80)}{12^2} \]

Hence, the sample size was calculated as 43. Accounting for a 10% attrition rate, the final sample size was set at 50 participants.

Data collection procedure

A pre-anesthetic evaluation was conducted 24 hours before surgery. Participants received a comprehensive explanation of the study protocol during this assessment and provided written informed consent. Demographic information, including age, sex, body mass index (BMI), and comorbidities, was recorded. Preoperative medications included intravenous (IV) pantoprazole (40 mg) administered the night before surgery and ondansetron (4 mg IV) on the day of the procedure.

Upon arrival in the preoperative area, fasting status was confirmed, baseline vital signs were recorded, and an 18G intravenous cannula was inserted. Standard monitoring was initiated in the operating room, which included pulse oximetry, non-invasive blood pressure, and electrocardiography. Patients were pre-oxygenated with 100% oxygen for three minutes, followed by intravenous administration of midazolam (0.2 mg/kg) and fentanyl (2 mcg/kg) for sedation and analgesia. Anesthesia was induced with intravenous propofol (2 mg/kg), and neuromuscular blockade was achieved using atracurium (0.5 mg/kg IV).

Endotracheal intubation was performed under direct visualization of the vocal cords using a laryngoscope with an appropriately sized blade. Simultaneously, a second clinician conducted suprasternal ultrasonography using a linear probe. Correct endotracheal tube (ETT) placement was confirmed by the presence of a hyperechoic shadow (the “bullet sign”) and a hypodense circular structure. Esophageal intubation was indicated by the absence of these findings or the appearance of a “double tract sign.” The time required for suprasternal ultrasound confirmation was measured from ETT insertion to visualization of the bullet sign.

Immediately thereafter, the ultrasound probe was repositioned to the subxiphoid area to evaluate diaphragmatic movement, and the time to confirmation was similarly recorded. To minimize observer bias, intubation and ultrasonographic assessments were performed by two independent clinicians, each blinded to the other’s findings. Ultrasound assessments were performed using a Sonosite M-Turbo portable ultrasound system (FUJIFILM Sonosite Inc., Bothell, WA). A linear array transducer (HFL38, 13-6 MHz) was utilized for suprasternal imaging, while a curvilinear probe (C60, 5-2 MHz) was employed for the subxiphoid evaluation. Imaging parameters were standardized across all scans: depth was set to 3-5 cm for the suprasternal view and 8-12 cm for the subxiphoid view, gain was adjusted individually to optimize soft tissue contrast, and the dynamic range was fixed at 60 dB. The probe was placed transversely at the suprasternal notch to obtain the suprasternal view, allowing visualization of the tracheal air shadow and identification of the hyperechoic “bullet sign.” The subxiphoid view focused on diaphragmatic motion, which was evaluated using M-mode with the cursor aligned over the right hemidiaphragm. All ultrasound scans were conducted by anesthesiologists with at least six months of focused airway ultrasonography experience and held certification from institutional point-of-care ultrasound (POCUS) training programs. Two independent operators were involved in each case: one was responsible for the endotracheal intubation, and the other performed the ultrasound examination. Both operators were blinded to each other’s procedures and findings to minimize observer bias and ensure objective assessment. A stopwatch was employed to document key time intervals with precision. The suprasternal ultrasound time was defined as the interval from endotracheal tube (ETT) insertion to the appearance of the “bullet sign.” The subxiphoid ultrasound time was recorded from the placement of the probe to the first visible diaphragmatic excursion, and capnography time referred to the duration from ETT insertion to the appearance of six consecutive capnographic waveforms. In contrast, auscultation time was calculated from ETT insertion to the completion of five-point auscultation. All these measurements were recorded to the nearest 10th of a second by a third observer, who remained blinded to both operators’ assessments.

**Figure 1:**
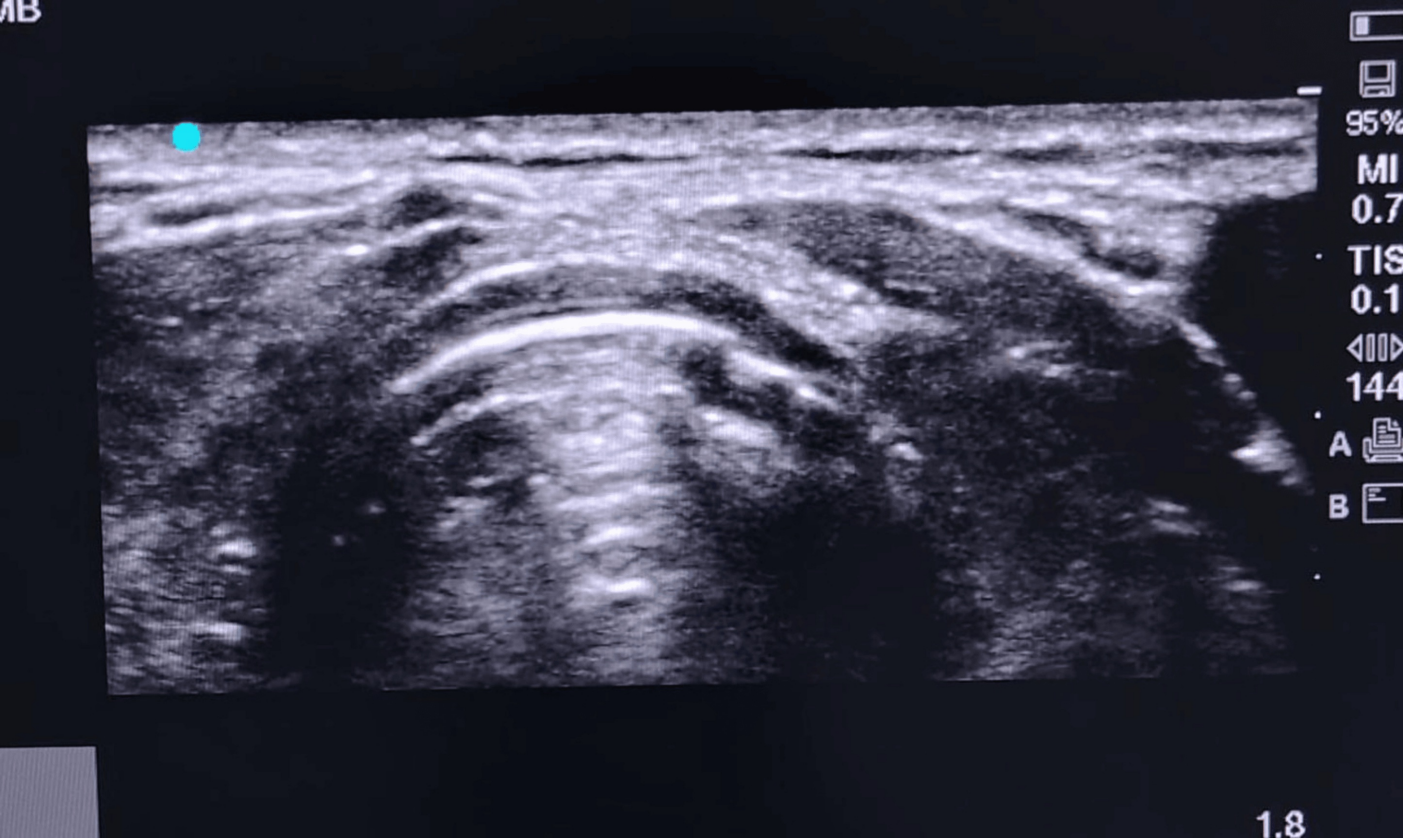
Suprasternal view The image demonstrates the trachea centrally, appearing as a hypoechoic rounded structure with posterior reverberation artifacts (comet tail), characteristic of air. The right and left thyroid lobes are seen laterally, appearing as homogeneous, moderately echogenic structures. The strap muscles are visualized anterior to the thyroid lobes as hypoechoic bands. This view is commonly used in thyroid and neck sonography for structural assessment. This original image was acquired directly from patients who participated in the study.

**Figure 2:**
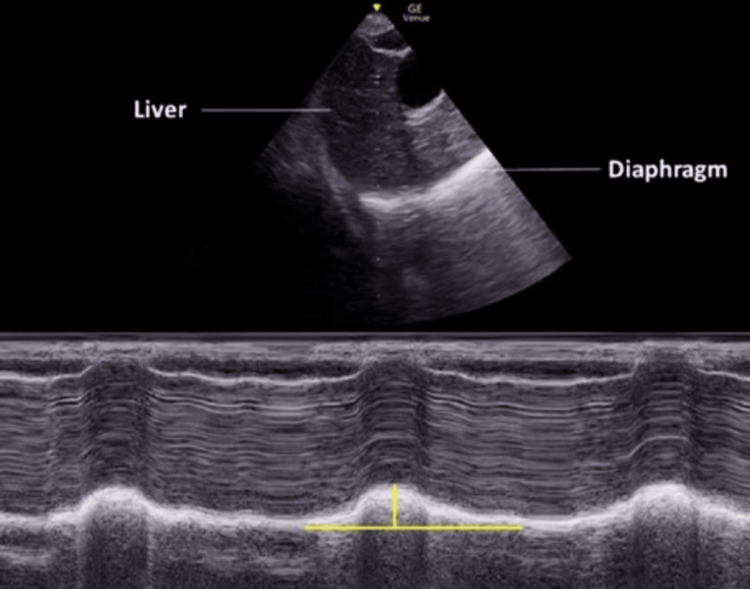
Subxiphoid view The upper panel shows a B-mode image with the liver and right hemidiaphragm labeled. The M-mode cursor is placed over the diaphragm to assess its movement during respiration. The lower panel displays the corresponding M-mode tracing, demonstrating diaphragmatic excursion as a sinusoidal wave pattern. The vertical yellow line represents the amplitude of diaphragmatic excursion during inspiration, which can be quantitatively assessed to evaluate diaphragmatic function. This technique is commonly used in the assessment of diaphragmatic paralysis or dysfunction. This original image was acquired directly from patients who participated in the study.

Additional confirmation methods included direct visualization of vocal cord passage, capnography (demonstrating six consecutive waveforms), and five-point auscultation. Continuous waveform capnography served as the reference standard, with the time to confirmation measured from ETT insertion to the appearance of six consistent capnographic waveforms (Figure 1 and Figure 2).

Following the confirmation of appropriate ETT placement, mechanical ventilation was initiated, and institutional protocols maintained anesthesia. Intraoperative hemodynamic parameters were continuously monitored. After surgery, neuromuscular blockade was reversed with intravenous neostigmine (0.05 mg/kg) and glycopyrrolate (0.01 mg/kg). Patients were extubated once standard recovery criteria were met, observed in the post-anesthesia care unit for 15 minutes, and transferred to the ward.

Statistical analysis

Data were analyzed using SPSS version 22 (IBM Corp., Armonk, NY) and Epi Info version 7.2.1 (Centers for Disease Control and Prevention, Atlanta, GA). The chi-square test was used to assess the significance of qualitative data. Continuous data were expressed as mean and standard deviation (SD). The normality of continuous variables was tested using the Kolmogorov-Smirnov test, and the t-test was used to analyze parameters with constant measurements.

## Results

The study population comprised 50 patients with a mean age of 41.86 years (±13.59 SD), indicating a predominantly middle-aged cohort. The majority were female patients, with 32 (64%) female patients and 18 (36%) male patients. Based on the ASA-PS classification, 27 (54%) were ASA 1, and 23 (46%) were ASA 2 (Table 1).

**Table 1:** Demographic data of the subjects ASA: American Society of Anesthesiologists

Variables	Number (%)
Gender	
Female	32 (64%)
Male	18 (36%)
ASA grade	
1	27 (54%)
2	23 (46%)

Preoperative airway assessment revealed that all patients (n = 50, 100%) had a standard mouth opening of at least 3 cm. The upper lip bite test (ULBT) classified 18 (36%) subjects as grade 1, suggesting moderate difficulty in some cases. The Mallampati grading was evenly distributed, with 25 (50%) classified as grade 1. These findings indicate that while no extreme airway difficulty was anticipated, a subset of patients presented with anatomical variations that could influence intubation (Table 2).

**Table 2:** Grades of assessment

Parameters	Number (%)
Mouth opening (3 cm)	50 (100%)
Upper lip bite test	
Grade 1	18 (36%)
Grade 2	32 (64%)
Mallampati grade	
1	25 (50%)
2	25 (50%)

The study population’s anthropometric measurements revealed normal ranges for adult airway anatomy, suggesting no significant morphological predictors of difficult intubation in the cohort (Table 3).

**Table 3:** Distribution of other measurements SD: standard deviation

Measurements	Mean ± SD
Neck circumference (cm)	33 ± 2.4
Thyromental distance (mm)	74.2 ± 3.8
Sternomental distance (mm)	134 ± 5.31
Thyromental height test (mm)	58.6 ± 3.5

Confirmation of endotracheal tube placement was achieved in 50 (100%) cases by suprasternal ultrasonography, 50 (100%) by subxiphoid ultrasonography, and 50 (100%) by continuous capnography (Table 4). Both suprasternal and subxiphoid ultrasonography correctly identified all 50 cases of proper tracheal intubation, matching perfectly with the gold standard of continuous capnography. This perfect concordance between ultrasound techniques and capnography (p-value not applicable due to 100% agreement) indicated that either ultrasound approach could reliably confirm endotracheal tube placement in this patient population. The absence of any false negatives or positives in the current study suggests that these ultrasound methods may be particularly effective in patients with normal airway anatomy and without obesity, as reflected in the inclusion criteria (Table 4).

**Table 4:** Suprasternal ultrasonography, subxiphoid ultrasonography, and continuous capnography wave findings

Parameters	Number (%)
Suprasternal ultrasonography	
Positive	50 (100%)
Negative	0 (0%)
Subxiphoid ultrasonography	
Positive	50 (100%)
Negative	0 (0%)
Continuous capnography waves	
Positive	50 (100%)
Negative	0 (0%)

Suprasternal ultrasonography demonstrated the shortest confirmation time, with a mean of 5.58 ± 1.1 seconds (95% CI: 5.25-5.91), suggesting superior efficiency in rapid verification of tube placement. Subxiphoid ultrasonography required a mean of 20.32 ± 4.5 seconds (95% CI: 19.01-21.63), while capnography and five-point auscultation took considerably longer, with mean times of 31.50 ± 4.8 seconds (95% CI: 30.13-32.87) and 33.38 ± 4.5 seconds (95% CI: 30.13-32.87), respectively. These findings indicate that suprasternal ultrasonography may offer a more time-efficient alternative to traditional methods for confirming endotracheal tube placement in clinical settings (Table 5).

**Table 5:** Comparison of time taken for ETCo2 results and other measurements The overall ANOVA was significant (p < 0.001), and pairwise comparisons were performed using independent samples t-tests based on reported means and standard deviations in Table 6. ETCo2: end-tidal carbon dioxide, ANOVA: analysis of variance, SD: standard deviation

Parameters	Total number	Mean ± SD	95% confidence interval
Time taken for capnography results	50	31.50 ± 4.8	30.13-32.87
Time taken for suprasternal ultrasonography (seconds)	50	5.58 ± 1.1	5.25-5.91
Time taken to confirm tube position by subxiphoid ultrasonography (seconds)	50	20.32 ± 4.5	19.01-21.63
Time taken for five-point auscultation	50	33.38 ± 4.5	30.13-32.87

**Table 6:** Pairwise comparison of time taken to confirm endotracheal tube position between different methods * represents a significant p-value. p<0.05 was considered significant. USG: ultrasonography

Pairwise comparison	t-value	p-value
Capnography versus suprasternal USG	33.3	<0.001^*^
Capnography versus subxiphoid USG	13.1	<0.001^*^
Capnography versus auscultation	2.0	0.048^*^
Suprasternal USG versus subxiphoid USG	18.5	<0.001^*^
Suprasternal USG versus auscultation	34.8	<0.001^*^
Subxiphoid USG versus auscultation	13.8	<0.001^*^

Pairwise comparisons were conducted to evaluate differences in the time taken to confirm endotracheal tube position between four methods: capnography, suprasternal ultrasonography (USG), subxiphoid USG, and five-point auscultation. Independent samples t-tests showed statistically significant differences between all pairs. The most considerable differences were observed between suprasternal USG and auscultation (t = 34.8, p < 0.001) and between capnography and suprasternal USG (t = 33.3, p < 0.001). The comparison between capnography and auscultation yielded a t-value of 2.0, corresponding to a borderline significant p-value of 0.048. All other pairwise comparisons demonstrated highly significant differences (p < 0.001). These results indicate that each method significantly differs in the time required to confirm tube placement (Table 6).

The diagnostic accuracy of suprasternal and subxiphoid ultrasonography for confirmation of endotracheal tube position was 100%, with a sensitivity of 100% (95% CI: 92.86-100) and a positive predictive value of 100% (95% CI: 92.86-100) (Table 7).

**Table 7:** Diagnostic performance of suprasternal and subxiphoid ultrasonography for endotracheal tube confirmation compared to continuous capnography CI: confidence interval

Parameter	Estimate	Lower-upper 95% Cis
Sensitivity	100%	92.86-100
Positive predictive value	100%	92.86-100
Diagnostic accuracy	100%	92.86-100

## Discussion

The present study’s demonstration of 100% diagnostic accuracy for suprasternal and subxiphoid ultrasonography in confirming endotracheal tube (ETT) placement represents a significant advancement in airway management. These findings align with capnography, the current gold standard, and reinforce the growing body of evidence supporting ultrasound as a first-line confirmatory tool [1,2]. While previous studies, such as that by Mousavi et al. (2022) [9], reported slightly lower sensitivities (98.5% for suprasternal and 96.2% for subxiphoid), the current results suggest that under optimal conditions, specifically, in non-obese patients with normal airway anatomy, ultrasound can achieve perfect concordance with capnometry.

The findings related to time efficiency are particularly noteworthy. In this study, suprasternal ultrasound confirmed ETT placement in just 5.58 ± 1.14 seconds, significantly faster than capnography (31.50 ± 4.84 seconds) and auscultation (33.38 ± 4.58 seconds). This rapid confirmation addresses a critical need in emergency airway management, where every second is vital [3]. These results are consistent with Sahu et al., who reported a 78% reduction in time to confirmation using ultrasound compared to capnography in emergency department settings [10]. Similarly, a recent systematic review by Patel et al. concluded that point-of-care ultrasound (POCUS) could reduce the incidence of unrecognized esophageal intubation in emergency cases by up to 92% [11].

The meta-analysis by Chen et al. (2022) reported slightly lower pooled sensitivity (97%), likely due to the inclusion of more heterogeneous patient populations [4]. The stringent exclusion criteria in the current study (BMI > 30 kg/m² and anticipated difficult airway) may explain the observed perfect accuracy. This finding is supported by Zhang et al., who demonstrated reduced ultrasound accuracy (91%) in obese patients (BMI > 35 kg/m²), attributed to poorer image quality [12]. Similarly, a multicenter ICU trial by Garnacho-Montero et al. (2024) reported 94% subxiphoid accuracy, further highlighting how patient factors influence diagnostic reliability [2]. Lee et al. (2024) also observed reduced sensitivity (89%) in patients with pre-existing lung pathology [13].

The suprasternal approach offers the advantage of direct visualization of the trachea and ETT, virtually eliminating false positives due to esophageal intubation, an issue more common with indirect confirmation methods [5]. This is particularly relevant considering NEAR III registry data indicating that 2.3% of emergency intubations result in unrecognized esophageal placement [14]. However, as noted by Myatra et al., the suprasternal technique may be technically challenging for patients with a short neck or cervical spine immobilization [15]. Conversely, while the subxiphoid view is more universally applicable, it relies on diaphragmatic and lung movement, making it less reliable in mainstem intubation, pneumothorax, or pleural effusions [16]. The current study’s finding of 100% accuracy in both methods within a carefully selected population highlights the importance of operator experience and appropriate patient selection, an observation echoed in the Society of Critical Care Medicine’s guidelines on adult critical care ultrasonography [17].

Emerging technologies may further enhance these techniques. Recent work by Liu et al. demonstrated that artificial intelligence-assisted ultrasound interpretation can reduce novice operator error rates from 15% to 3% [18]. Moreover, developing hybrid probes combining linear and phased-array capabilities could enable simultaneous suprasternal and subxiphoid assessment, potentially leveraging the advantages of both approaches [19].

Limitations

This study has several limitations that merit consideration. First, the research was conducted at a single tertiary care center, which may introduce selection bias and limit the generalizability of our findings to broader clinical settings with varying patient populations and institutional protocols. Second, we excluded patients with a body mass index (BMI) > 30 kg/m² and those with anticipated difficult airways. While this approach reduced variability and ensured image quality, it also limits the applicability of our results to high-risk populations, such as obese individuals or patients with anatomical airway challenges, where ultrasound may perform differently. Third, no cases of esophageal intubation occurred in our cohort, precluding the estimation of specificity and negative predictive value. This likely reflects the elective nature of the surgeries and the exclusion of patients at risk for difficult intubation. As a result, while sensitivity and positive predictive value were calculated, our diagnostic accuracy metrics cannot be fully generalized to emergency or high-risk scenarios. Fourth, while our study employed blinded dual-clinician assessments to reduce observer bias, we did not report inter-operator reliability metrics, such as kappa statistics or intraclass correlation coefficients, which could quantify the consistency of ultrasound interpretation across different users. Additionally, we did not account for variability in operator training; although all sonographers had a minimum of six months of focused airway ultrasound experience, the absence of formal standardized certification across centers may affect reproducibility in other settings.

Future research should aim to validate these findings in more diverse populations, including obese, pediatric, and trauma patients. Multicenter trials would enhance generalizability, while developing standardized training protocols may reduce inter-operator variability. Additionally, integrating artificial intelligence for real-time ultrasound interpretation could improve diagnostic accuracy and broaden accessibility in resource-limited settings.

## Conclusions

In conclusion, ultrasonography is a practical and efficient tool for confirming endotracheal tube (ETT) placement in clinical settings. The suprasternal and subxiphoid approaches each offer distinct advantages: direct trachea visualization and diaphragmatic movement assessment, respectively. These methods are practical when rapid and reliable airway confirmation is essential in time-sensitive situations. While the techniques show strong potential, further research across varied patient populations and clinical environments is necessary to support their broader adoption in routine airway management.
